# Surgical palliation in poorly differentiated neuroendocrine carcinoma of the hypopharynx: Case report

**DOI:** 10.1002/cnr2.1558

**Published:** 2021-10-05

**Authors:** Francesca Romana Fiorini, Yasmin Abbas, Suchana Mukhopadhyay, Taran Tatla

**Affiliations:** ^1^ Departmentof Otolaryngology and Head and Neck Northwick Park Hospital London UK

**Keywords:** hypopharynx, neuroendocrine carcinoma, surgical palliation

## Abstract

**Background:**

Primary neuroendocrine carcinomas (NECs) are very rare entities accounting for 0.49% of all malignancies. Within the head and neck, the most common sites are the larynx and paranasal sinuses, while the hypopharynx is seldom described.

**Case:**

We present a patient with a poorly differentiated metastatic NEC of the hypopharynx treated palliatively with organ‐preserving surgery and post‐operative chemotherapy, and literature review for well‐documented pure hypopharyngeal NECs. Our patient died of chest infection during chemotherapy, 4 months after surgery.

**Conclusion:**

Chemotherapy remains the mainstay of treatment in the presence of metastases with 2‐year overall survival of 15.7%. Due to the aggressive nature of poorly differentiated metastatic NECs, surgical management is seldom considered. We report and advocate the successful palliative role of organ‐preserving, minimally invasive trans‐oral LASER micro‐surgery and neck dissection to control loco‐regional head and neck disease, safe‐guarding better quality of home life, despite limited life expectancy for this condition.

## 1 INTRODUCTION

Primary neuroendocrine carcinomas (NECs) are very rare entities accounting for 0.49% of all malignancies. Historically, NECs have been recognised as arising from the gastrointestinal tract and pulmonary system,^1^ but there is increasing evidence of their origin from other anatomical regions, including the head and neck,^2^, ^3^, ^4^ where the most common sites represented are the larynx and paranasal sinuses.

Recently, the 2017 WHO Classification of Head and Neck Tumours identified four groups: well differentiated, moderately differentiated and poorly differentiated (the latter with small cell and large cell types).^5^, ^6^ This new classification closely correlates to the 5‐year disease‐specific survival (DSS) of 100, 52.8, 19.3 and 15.3% for each diagnostic group.^7^

Literature supports the evidence that the best treatment available for poorly differentiated NECs of the larynx is platinum‐based chemotherapy with concomitant radiotherapy, although surgery alone has also been proposed, as well as surgery followed by chemo‐radiotherapy (CRT).^7^, ^8^ The limited number of patients presenting with a NEC of the hypopharynx have seemingly been treated with multimodal therapy, achieving differing outcomes..^9^, ^10^, ^11^, ^12^, ^13^, ^14^, ^15^, ^16^, ^17^, ^18^, ^19^, ^20^, ^21^, ^22^, ^23^

In the present article, we describe a case of small cell poorly differentiated metastatic NEC of the hypopharynx. We underline the practical benefits of a non‐radical, palliative surgical approach to ensure a better quality of home and family life in a disease with a very poor prognosis. A literature review is also presented.

## 2 CASE

A 61‐year‐old woman was referred to the Head and Neck Department of a Tertiary Care Centre in London for a 2 months history of progressive dysphagia, change in voice and enlarging right cervical lymphadenopathy. She was an ex heavy smoker (40 pack/year) who quit 12 months earlier, with a history of 6 units of alcohol per week. She suffered from hypertension, hypercholesterolaemia and epilepsy, all well controlled on medication.

Clinical examination showed a large, expanding conglomerate nodal mass at right neck level II to IV with palpable contralateral cervical disease. Fiberoptic laryngeal examination showed an exophytic lesion obliterating the whole right pyriform fossa, spilling over marginally onto the right aryepiglottic fold and laryngeal vestibule; although reduced right vocal cord motility, the airway was uncompromised.

**FIGURE 1:**
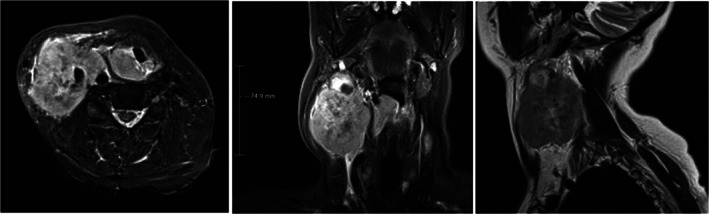
T2‐weighted magnetic resonance imaging head and neck sequences (axial, coronal and sagittal in sequence) showing a 3.5 x 3.4 x 1.6 cm mass centred in the right pyriform fossa, involving the hypopharyngeal surface of the right aryepiglottic fold, extending posteriorly and inferiorly into the hypopharyngeal wall where it crossed the midline. Laryngeal cartilages and larynx itself spared from within. The right level II to IV lymph node mass measured up to 7.5 cm in craniocaudal diameter, encasing the common carotid artery to its bifurcation and obliterating the IJV; the left level III lymph node measured 2.3 cm and level II 1.4 cm

An magnetic resonance imaging (MRI) scan of the neck with contrast (Figure 1) showed a 3.5 x 3.4 x 1.6 cm mass centred in the right pyriform fossa and a right level II to IV large lymph node conglomerate mass measuring up to 7.5 cm in craniocaudal diameter, encasing the common carotid artery to its bifurcation and obliterating the IJV. Separate abnormal left level II lymph and level III lymph nodes measuring 1.4 and 2.3 cm respectively were noted.

A^68^Ga‐DOTATATE PET‐CT showed pulmonary nodules (non‐avid for tracer), highly suspicious for metastases, with primary right hypopharynx mass and bilateral cervical lymph nodes (right > left; all non‐avid for tracer). The patient was radiologically staged as T4aN3bM1 (stage IVC).

**FIGURE 2:**
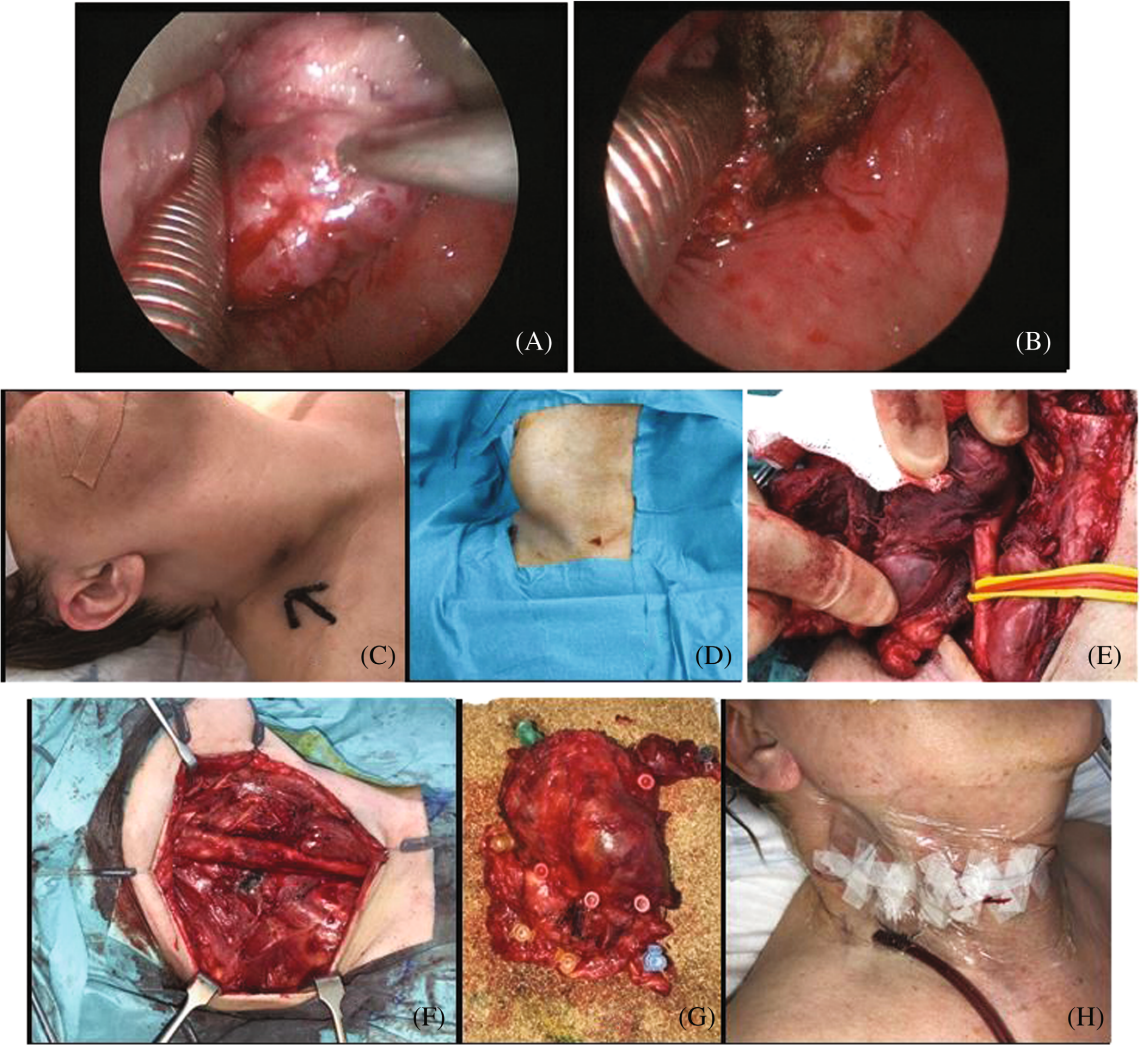
Intraoperative pictures: exophytic lesion in the right pyriform fossa before (A) and after (B) CO_2_ LASER debulking; (C–E) right neck mass, encasement of the CCA noticeable; (f) image of the neck following RND, the mass was peeled off the CCA, which was fully preserved; (g) neck dissection specimen pinned on cork board; (h) appearance of the neck after closure of the wound. CCA, common carotid artery; IJV, internal jugular vein; RND, radical neck dissection

We performed a panendoscopy and biopsy of the hypopharynx mass, which revealed a small cell poorly differentiated NEC. Immunostains showed patchy positivity for AE1/AE3 and widespread positivity for CAM5.2 and CD56. Very few cells expressed CK5, while they were negative for p63, thyroglobulin, synaptophysin, chromogranin, TTF1, CK7, CK20 and Napsin A. After formal Head and Neck Cancer MDT discussion (and debate on the advantages and disadvantages of radical laryngo‐pharyngectomy), in agreement with patient and family, we performed a trans‐oral CO_2_ LASER debulking of the primary tumour along with a right radical neck dissection (sternocleidomastoid muscle, IJV and accessory nerve sacrificed, Figure 2) for loco‐regional disease and symptom control, allowing chemoradiation to then be progressed at a neighbouring quaternary referral unit hosting a super‐specialist NEC MDT. Nutritional support was ensured via a percutaneous endoscopic gastrostomy tube in the immediate perioperative period, however she made a good recovery and was restarted on a soft diet a few days after surgery. She was discharged home 11 days post‐operatively.

Final histology confirmed a small cell poorly differentiated NEC, 2 out of 20 right cervical lymph nodes showing metastatic carcinoma, with the largest tumour deposit of 100 mm (macroscopic measurement) involving right level II, III and IV with extracapsular spread. Lymphovascular and perineural permeation was present. The tumour showed diffuse strongly positive staining for AE1/3, CAM 5.2, CD 56 and patchy strong staining for chromogranin and synaptophysin. P63 and CK5 were negative. The CK5 negative profile in the lymph node tissue allowed the pathologist to conclude that CK5 positivity in the hypopharyngeal (pyriform fossa) biopsies was likely native squamous epithelium.

A restaging FDG PET‐CT 6 weeks following surgery showed metastatic disease in the liver and lung with pleural involvement, although she was asymptomatic from this. She received post‐operative chemotherapy including three cycles of platinum/etoposide doublet therapy, along with zolendronic acid infusion to control tumour‐induced hypercalcemia.

At her last follow‐up appointment, 8 months after first GP presentation, her airway remained uncompromised despite obvious clinical disease progression (smaller volume recurrence in right neck and at primary hypopharynx site). Through trans‐oral CO_2_ LASER debulking, she avoided the multiple limitations associated with tracheostomy care and continued to manage a soft diet and oral liquids safely, using the gastrostomy tube feed to supplement her nutrition. Her pain was overall well controlled. The palliative radical neck dissection was associated with immediate relief of pain and discomfort caused by grossly enlarged and bulky unilateral cervical lymph nodes that limited neck movements. This nodal enlargement also threatened skin thinning and tumour ulceration, as well as provided a significant source of social embarrassment for the patient impacting her mental health, due to a grossly distorted appearance for neck and facial morphology.

Although she was offered FOLFIRI (folinic acid, fluorouracil and irinotecan) second line chemotherapy, unfortunately she developed a chest infection requiring hospitalisation. She died of presumed cardiopulmonary arrest, before receiving this, a full 4 months following palliative surgery. There was a 2 months delay from initial presentation to ENT to commencement of definitive palliative treatment due to the care pathway limitations associated with referring patients through three separate regional cancer MDTs from Watford District General Hospital. Initial discussions held at head and neck cancer MDT at London North West, then at Imperial, and then finally Royal Free Hospital NEC MDT, the three MDTs being located at separate hospitals in Northwest, West and North London.

## 3 DISCUSSION

Poorly differentiated NECs of the head and neck are a rare disease entity; among them, those arising from the hypopharynx represent the most uncommon subsite origin. Similar to hypopharyngeal squamous cell carcinomas, these cancers often remain silent until they start compromising swallowing and breathing, that is, locally enlarge to impact the endoluminal calibre, or spread to cervical lymphnodes and distant organs.

The 2017 WHO Classification of Head and Neck Tumours identified four groups of NECs: well differentiated, moderately differentiated and poorly differentiated (the latter with small cell and large cell types).^5^, ^6^ The curves of 5‐year DSS drop dramatically from the second to the third and fourth group, from 52.8 to 19.3 and 15.3%,^7^ underlining the aggressiveness of poorly differentiated NECs.

There is a number of retrospective studies in the literature^2^, ^3^, ^4^, ^8^ focusing on NECs of larynx, which remains the most common site of presentation for this rare entity in the head and neck region.

In the largest meta‐analysis by Van der Laan et al,^7^ which includes 436 reported cases of laryngeal NECs, CRT appeared to be the best management option for small cell NECs, with increased 5‐year DSS compared to other modalities (31 vs. 13%, *p* = .001).

Conversely, current literature includes only a limited number of pure hypopharyngeal NECs.^9^, ^10^, ^11^, ^12^, ^13^, ^14^, ^15^, ^16^, ^17^, ^18^, ^19^, ^20^, ^21^, ^22^, ^23^, ^24^, ^25^ Table 1 summarises their main characteristics.

**TABLE 1:** Characteristics of pure hypopharyngeal NECs in published English literature

Author[ref] year	Number of cases	Age/sex	Subsite	TNM/stage	Main symptoms	Treatment	Follow‐up time (month)	Follow‐up status
Baugh,^20^1986	1	63, F	PS	T4aN2cM0, stage IVA	Dysphagia, cervical lymphadenopathy	Non radical surgery, multiple courses of CHT	55	Disease free
Gaba,^10^ 2005	1	65, M	PS	T4aN1M0, stage IVA	Dysphagia, weight loss	CHT (platinum‐based) and RT	24	Disease free
Okuda,^13^ 2005	1	72, M	Nasopharynx, hypopharynx	T4N2cM0, stage IVA	Pharyngodinia and dyspnoea	CHT (cisplatin, etoposide) and RT	24	Alive with disease
Sano,^14^ 2005	1	67, F	PS	NA	Cervical lymphadenopathy	Multiple courses of CHT (etoposide, carboplatin) and RT	13	Deceased of lung and liver mets
Yoshida,^21^ 2005	1	78, M	PS	T2N0M0, stage II	Pharyngodinia	CHT (docetaxel, cisplatin, 5‐fluorouracil) and RT	36	Disease free
Takagawa,^15^ 2011	1	59, M	NA	NA (T unknown at 1^st^presentation, liver mets 16 months after)	Cervical lymphadenopathy	Radical surgery, multiple courses of CHT and RT (to primary and mets)	39	Deceased of liver mets
Bayram,^22^ 2015	1	50, M	PS	T4bN2bM1, stage IVC	Pharyngodinia, cervical lymphadenopathy	CHT (etoposide, cisplatin) and RT	15	Disease free
Lee‐WI,^16^ 2015	1	56, M	PH	T4aN3M1, stage IVC	Dysphagia, hoarseness, cervical lymphadenopathy	CHT (platinum, etoposide)	NS (weeks)	Deceased of cardiopulmonary arrest
Pointer^a^,^26^ 2017	34	60 (median), M:F 2:1	NA	Stage IVA most common (32.4%)	NS	Stage related	Stage related	Stage related
Sun,^23^ 2018	1	NA F	PH	NS	Fluctuating cervical lymphadenopathy	CHT (cisplatin, etoposide) and RT	3	Disease free
Ao,^24^	1	66, M	PH	T2N2M0	Throat pain, hoarseness, dysphagia	CHT + RT	7	Deceased of multiple mets

Abbreviations: CHT, chemotherapy; mets, metastases; NA, not available; NECs, neuroendocrine carcinomas; PH, posterior hypopharynx; PS, pyriform sinus; RT, radiotherapy.

^a^ See Table 2 for more information.

These tumours often present as loco‐regionally advanced disease, that is, with cervical lymphadenopathies and more than 90% develops distant metastases.^26^ Lee et al^16^ described a case presenting with bone, liver and lung metastases, who died a few weeks after the second cycle of chemotherapy from presumed cardiopulmonary arrest. Takagawa et al^15^ presented a patient who received neck dissection and multiple courses of CRT to the primary and to lung and bone metastases, who deceased 39 months after surgery. Bayram's patient^22^ had lung metastases at presentation but responded well to CRT, reported disease‐free 15 months after. Sano et al^14^ achieved ‘complete response’ at primary site to CRT in a case of primary small cell carcinoma of the hypopharynx, however the patient died of lung and liver metastases 1 month after treatment. In the presence of distant metastases, the outcomes remain poor no matter what treatment is given.

In addition to these anecdotic cases, Pointer et al^26^ published an analysis of the US National Cancer Database, which focused on poorly differentiated and small cell carcinoma of the head and neck in a well‐defined time‐frame. To date, this is the largest published retrospective series of head and neck NECs with 34 recorded patients presenting with hypopharyngeal localization. Hypopharynx was again the less frequent site of NEC, accounting for only 4% of the whole head and neck population: one third of these cases were locally advanced and one fifth metastatic disease (stage IVC).

In the metastatic group, chemotherapy alone or a combination of CRT was the most common treatments administered. The presence of distant metastases clearly worsened prognosis with a median survival of 10 months and 2‐year overall survival of 15.7%. Interestingly, combining radiotherapy and chemotherapy did not result in improved survival (*p* = .14), confirming the aggressive nature of the disease (Table 2).

**TABLE 2:** Characteristics of hypopharyngeal NECs in the analysis of the US National Cancer Database^19^

Number of cases	34
Stage I	2 (5.9%)
II	1 (2.9%)
III	10 (29.4%)
IVA	11 (32.4%)
IVB	3 (8.8%)
IVC	7 (20.6%)
Treatment by stage	See text
Median overall survival (months) by stage (Larynx/Hypopharynx)	
Stage I/II	29.1
III/IV/IVB	19.1
IVC	10.2
2‐year overall survival	
Stage I/II	65.3%
III/IV/IVB	42.3%
IVC	14.2%

Abbreviations: NECs, neuroendocrine carcinomas.

Our patient developed loco‐regionally advanced disease that impacted her swallowing, as well as asymptomatic lung disease; therefore, she already had stage IVC NEC at first presentation. After analysing all available data, we concluded that offering radical surgery to the primary (as one would debate with SCC of the hypopharynx, i.e., laryngopharyngectomy), would not be in her best interest.

For disease associated with such poor prognosis, one should keep paramount the importance of achieving symptom‐control for quality of life, rather than prolonged survival. The paucity of data did not allow for definitive conclusions on therapy, allowing merits and risks for all options to be considered.

Roland and Bradley^27^ point out the role of surgery as a palliative treatment in head and neck cancer. The choice of treatment in terms of morbidity has a key role, as intervention may impact on swallowing and speech, breathing, mastication and appearance. Therefore, when discussing the available options, minimally invasive surgical palliative care should be considered to improve both quality of remaining life and quality of dying, by reducing the burden of existing symptoms and preventing the onset of new ones.

The diagnostic workup of NEC has always been challenging. For instance, because of the large variability for tumours in proliferation rate (Ki67) and SSR (somatostatin receptor) subtype profile, no single modality is entirely effective. A combination of anatomic and functional techniques is routinely performed to optimise sensitivity and specificity (overall ∼80–90%).^28^

The widespread use of ^68^Ga‐labelled octreotide derivatives DOTATOC, DOTATATE and DOTANOC (^68^Ga‐SSA‐PET‐CT)^29^ has significantly increased overall sensitivity to >90%, while specificity ranges between 92 and 98%. The main limitation appears lack of reproducibility due to the lack of standardisation regarding preparation, production procedure and examination protocols.

Studies have also compared ^68^Ga‐DOTANOC to standard imaging (CT, MRI, US, OctreoScan).^30^, ^31^ In metastases,^68^Ga‐DOTANOC had a sensitivity of 97.4%, while conventional imaging a sensitivity of 81.8%, in particular for detection of lymph node metastases (*p* < .0001). In our case, the pre‐operative DOTATATE PET‐CT staging and then post‐operative FDG PET‐CT restaging scans both provided useful information, albeit limitations for contrasting metastatic nodules.

The hypopharyngeal biopsies were not reactive for chromogranin or synaptophysin, positive only for CD56 (a sensitive but less specific marker for neuroendocrine differentiation than chromogranin and synaptophysin). Furthermore, there were few CK5 positive cells (marker for squamous differentiation). Our pathologists interpreted this as displaced surface native squamous epithelium.

In the tissue microarray study by Lewis et al,^32^ neuroendocrine markers are only rarely expressed by head and neck SCC, while the squamous specific markers p40 and CK 5/6 are very sensitive for squamous differentiation and indeed expressed in the vast majority of head and neck SCC, and SCC with neuroendocrine marker expression. In the tissue microarray study, chromogranin‐A, synaptophysin and CD56 are highly specific neuroendocrine immunohistochemistry markers.

The final histopathology showed diffuse strongly positive staining for AE1/3, CAM 5.2, CD 56 and patchy strong staining for chromogranin and synaptophysin. P63 and CK5 were negative, hence our pathologists were able to confirm the diagnosis of NEC.

We agreed to minimally invasive debulking surgery of the primary to secure airway safety and avoid tracheostomy, as well as allow continuation of oral swallow following therapist assessment. The right radical neck dissection improved cosmesis and pain through removal of large neck metastases. As the nodal mass had extracapsular spread, encasing the common carotid artery and directly invading adjacent laryngeal cartilages, intraoperative microscopic control of cervical disease was not possible. A percutaneous endoscopic gastrostomy allowed nutritional supplementation before and after surgery, although she safely restarted oral diet a few days post‐operatively without aspiration. The support of our multidisciplinary team facilitated pain control, safe swallow and voice rehabilitation enabling uneventful discharge 11 days following surgery.

When dealing with individuals with head and neck cancer the impact of RT, CHT or surgery on quality of life must be considered. Each treatment may have a significant impact on swallowing (particularly RT), speech (particularly surgery) and cause systemic toxicity (CHT), however those patients with suitable tumours and access to trans‐oral microsurgical resection appear to have the least negative effects.^33^

Moreover, the role of early and concurrent palliative care is essential and helps patients and their families understand the goal of any form of treatment including surgery, prepare for evolving events and understand the ambivalence linked to the management of advanced disease.^34^

Our patient continued her care in an ambulatory manner living with her family at home, travelling to and from the specialist NEC centre for CRT as an outpatient before succumbing to a chest infection and passing away. Her care was undisturbed by the burden of having to care for a tracheostomy (and its secondary impact on voice and swallow) and nursing a fungating, ulcerating neck nodal mass, as well as free of restrictions on neck and shoulder movements and social embarrassment resultant from such a large asymmetric disfiguring neck mass.

The 2010 North American Neuroendocrine Tumour Society Consensus guideline^35^ recommended platinum and etoposide as first‐line chemotherapy for metastatic high‐grade extrapulmonary NECs. In agreement with the literature, we offered three cycles of platinum and etoposide to our patient, although she did not respond with disease progressing at both primary and cervical sites. Despite this, however, she experienced no airway difficulties, managing to talk and swallow safely at home until her terminal event.

In summary, NECs of the head and neck represent a rare clinical entity with an aggressive nature. Poorly differentiated NECs have intrinsic metastatic potential that impacts significantly on survival. All such cases should be managed with appropriate expert input of specialists regularly managing NEC across organ systems including gastrointestinal and head and neck primary origin.

Despite the low incidence of hypopharyngeal NEC, few large‐scale studies and absence of reported randomised controlled trials, CHT alone or combined CRT are reported as the best available treatment options.

Where loco‐regional disease bulk impacts (or may start to impact) upon mental health and basic human functions defining quality of life during palliation (ability to breathe, voice and swallow), organ‐preserving and minimally invasive surgical treatment provides an important treatment adjunct, necessitating key expertise of the ENT‐Head and Neck surgical team.

In this case, the social embarrassment of unaesthetic, gross and asymmetrically enlarged painful neck nodes with propensity for fungation was avoided through neck dissection. Transoral CO_2_ LASER debulk safeguarded the airway, avoided tracheostomy‐associated limitations for patient mobility and quality of life, allowing safe and unrestricted mobility at home during remaining months of life.

Transoral LASER surgery, accompanied by neck dissection where required, can play an important role controlling local symptoms and improving quality of life, as part of an informed and considered multi‐disciplinary palliative care plan.

## CONFLICT OF INTEREST

The authors declare there is no conflict of interest.

## AUTHOR CONTRIBUTIONS

All authors had full access to the data in the study and take responsibility for the integrity of the data and the accuracy of the data analysis. *Conceptualization*, T.T. and F.R.R.; *Validation*, Y.A.; *Investigation*, F.R.F.; *Writing—Original Draft*, F.R.F and T.T.; *Writing—Review & Editing*, T.T, Y.A and S.M; *Supervision*, T.T.

## ETHICAL STATEMENT

We obtained Institutional Review Board approval and the next of kin consent before disclosing the patient personal information.

## Data Availability

Data sharing not applicable to this article as no datasets were generated or analysed during the current study.
